# Theoretical Analyses of Turgor Pressure and Expansive Growth Rate of Plant Cells During Water Deficit

**DOI:** 10.3390/plants14223538

**Published:** 2025-11-20

**Authors:** Joseph K. E. Ortega

**Affiliations:** Department of Mechanical Engineering, University of Colorado Denver, Denver, CO 80217-3364, USA; joseph.ortega@ucdenver.edu

**Keywords:** turgor pressure, plant growth, expansive growth, water deficits, root growth, biophysical equations, dimensionless numbers

## Abstract

Expansive plant growth during water deficit is common in temperate and extreme climates. Understanding how the turgor pressure, *P*, behaves during water deficit is essential for a better understanding of expansive growth rate, *v*. Here, validated biophysical equations together with dimensional analyses are used to simulate water deficit and determine the behavior of *P* and *v*. A dimensionless number, Π_pw_, helps simplify the biophysical equations and interpret the results. The magnitude of Π_pw_ increases as water deficit severity increases. Analyses reveal that both *P* and *v* decrease curvilinearly as Π_pw_ increases. Simple mathematical relationships between *P*, *v*, and Π_pw_, are derived, providing a clear and quantitative understanding of how *P* and *v* change as water deficits become more severe. Additionally, it is shown how the results of these analyses can be used to assess *P* and *v* of roots growing in water deficit.

## 1. Introduction

Early in life we learn that water is required for plant growth. We learn that plants can grow when water is limited but not when it is absent. And we observe how plants that lack water become limp and less turgid. Of course, a much better understanding is needed to improve our ability to address many important problems such as crop production in adverse conditions and climate change [1]. With the introduction of new tools over the past century, a considerable amount of molecular and genetic research has been conducted to understand plant growth in water deficits, e.g., [2,3,4]. However, our understanding of the behavior and regulation of turgor pressure and expansive growth during water deficit is far from satisfactory. In a 1973 review, Hsiao [5] noted that “with the shift of attention to metabolic and molecular aspects of stress physiology in the mid-1960s, the importance of water uptake and the resulting turgor as a physical force needed for cell growth has at times been almost overlooked or ignored.” And in 2024 after quoting Hsiao [5], Voothuluru et al. [6] stated that “Arguably, the same statement could still be made today, and the role of turgor as well as mechanisms of turgor regulation and turgor sensing remain important areas for further investigation.” Other recent reviews echo the need for a better understanding of turgor pressure behavior and regulation in plant growth [7,8].

A problem in the pursuit of a better understanding is that water deficits can elicit many responses from plants, not all of which are directly relevant to growth. In addition, of those responses related to growth, some are biologically active, and others are biologically passive. In this study, biologically active responses are defined as those that change the biophysical properties of the organelles involved in expansive growth during water deficits, i.e., those responses that elicit a change in osmotic pressure, water transport properties of the plasma membrane, and mechanical properties of the cell wall. In contrast, biological passive responses are defined as those where the biophysical properties of the relevant organelles remain relatively constant during water deficits. The biological passive responses can be complicated and can appear to be biologically active responses. Therefore, it is important to have a good theoretical understanding of biological passive responses when water is limited, so that biologically active responses can be distinguished from biologically passive responses.

The overall objective of this study is to provide a *quantitative* understanding of biological passive responses of turgor pressure and expansive growth rate to water deficits and demonstrate how active responses to water deficits can be detected. The specific objective is to modify validated biophysical equations to reveal the relationship between the turgor pressure, expansive growth rate, and severity of water deficit, and then demonstrate how active changes in the biophysical properties of relevant organelles can be detected using these mathematical relationships.

### 1.1. Biophysical Perspective of Expansive Growth

Plants increase in size by permanently increasing the volume of their constitutive cells, i.e., expansive growth. From a biophysical perspective, two organelles are predominately involved in expansive growth, the plasma membrane and the cell wall. The plasma membrane encloses the protoplast and a wall chamber encloses the plasma membrane. The plasma membrane is a bilipid membrane embedded with a variety of large and small protein molecules, some of which are water channels (aquaporins) that make the membrane semi-permeable [9]. The wall chamber is composed of polysaccharide networks embedded with proteins [10]. Expansive growth requires the wall chamber to undergo irreversible (plastic) deformation in order to permanently increase the volume of the cell.

Turgor pressure, *P* (the pressure difference inside, *P*_i_, and outside, *P*_o_, of the plasma membrane; *P* = *P*_i_ − *P*_o_), provides the force for the wall deformation, ***F*** = *P**A*** (bold letters represent vectors). And water uptake by osmosis produces the *P*. In a model (ideal) nongrowing plant cell, the *P* is equal to the osmotic pressure difference, Δ*π* = *π*_i_ − *π*_o_. In a growing cell, wall stress relaxation reduces the *P* to produce water flow into the cell, i.e., water uptake. The rate of water uptake is related to the magnitude of the difference between Δ*π* and *P*, and the magnitude of the relative hydraulic conductance of the plasma membrane, *L*. The magnitude of *L* is related to the number of aquaporins in the plasma membrane (see Appendix A for definitions of variables and units).

*P* stresses the wall and produces reversible (elastic) deformation. When *P* exceeds a magnitude, *P*_C_ (critical turgor pressure), the wall deformation is both elastic and plastic. Plastic deformation of the wall is produced and regulated by breaking load-bearing bonds between wall polymers (wall loosening) using protein catalyst such as expansins and enzymes, and/or changing the pH in the wall [10]. The magnitude of the rate of plastic wall deformation is related to a variable termed the relative irreversible wall extensibility: *ϕ*. The rate of wall loosening is related to the magnitude of *ϕ*. An increase in *ϕ* reflects an increase in the rate of wall loosening and an increase in the rate of wall stress relaxation. The increase in wall stress relaxation decreases the *P*, which increases the rate of water uptake. Together, these processes result in an increase in the plastic deformation rate of the wall chamber and an increase in expansive growth rate, *v*. An analytic description of this process is presented in [11].

### 1.2. Biophysical Equations Describing Expansive Growth

A comprehensive understanding of a system and its behavior requires a detailed understanding of its individual components and a *quantitative* understanding of the relationship between the individual components. In the history of science, relevant interrelated equations, often called governing equations, have proven to be invaluable to a quantitative understanding of system behavior, e.g., Navier-Stokes’ equations in fluid mechanics [12] and Maxwell’s equations in electrodynamics [13]. Throughout the recent decades, biophysical equations that describe expansive growth rate of walled cells have been developed and can act as governing equations. Biophysical equations that describe the biophysical framework presented in the previous section have been derived and validated with experimental results from plant and fungal cells [14,15,16,17,18,19,20]. Although these equations have been reviewed [21,22], a short description of these equations is presented here for the reader’s convenience and for a better understanding of the analyses that follow. The biophysical equations presented here neglect the transpiration water loss from individual cells, which will be the subject of a subsequent theoretical study. The complete set of equations that include a term for transpiration water loss can be found in the following papers [18,19,20,21,22].

#### 1.2.1. Rate of Water Uptake

The rate of change in water volume encompassed by the plasma membrane as a function of time is d*V*_w_/d*t*. The rate of change in relative water volume is; *v*_w_ = (d*V*_w_/d*t*)/*V* = d*V*_w_/*V*d*t*, where *V* is the volume of the cell [14].

(1) $$v_{w}=L\;\left(∆\pi -P\right)$$

The *L* is related to the hydraulic conductivity, *L*_p_, of the plasma membrane, *L* = *L*_p_ (*A*/*V*) where *A* is the area of the plasma membrane. Equation (1) states that the relative water uptake rate, *v*_w_, is equal to the product of the hydraulic conductance, *L*, and the difference in the osmotic pressure difference, Δ*π*, and turgor pressure, *P*.

#### 1.2.2. Rate of Deformation of the Cell Wall

The rate of change in volume encompassed by the cell wall chamber as a function of time is d*V*_cw_/d*t*. The rate of change in relative volume enclosed by the wall chamber is; *v*_cw_ = d*V*_cw_/*V*d*t* [17].

(2) $$v_{cw}=\phi \;\left(P-P_{C}\right)+\left(\frac{1}{\varepsilon }\right)\;\frac{dP}{dt}$$

*ε* is the volumetric elastic modulus of the wall chamber. Equation (2) states that the relative deformation rate of the wall, *v*_cw_, is equal to the sum of the relative *plastic* deformation rate, $\phi \;\left(P\;-\;P_{C}\right)$, and the relative *elastic* deformation rate, $\left(\frac{1}{\varepsilon }\right)\;\frac{dP}{dt}$. Expansive growth rate of a plant cell requires that the relative plastic deformation rate of the wall is nonzero.

#### 1.2.3. Rate of Change in the Turgor Pressure

An equation describing the rate of change in turgor pressure, d*P*/d*t*, can be obtained by recognizing that *v*_w_ = *v*_cw_. Solving for d*P*/d*t*, Equation (3) is obtained [17].

(3) $$\frac{dP}{dt}=\varepsilon \;\left\{L\left(∆\pi -P\right)-\phi \;\left(P-P_{C}\right)\right\}$$

Equation (3) states that the rate of change of *P* is equal to the product of the volumetric elastic modulus, *ε*, and the difference in the relative water uptake rate, $L\left(∆\pi -P\right)$, and the relative plastic deformation rate of the wall, $\phi \;\left(P-\;P_{C}\right)$.

### 1.3. Dimensionless Numbers and the Physical Interpretation of the Variables

A dimensionless form of Equations (1)–(3) was obtained using dimensional analyses [23]. The coefficients of the dimensionless terms yield dimensionless groups of variables (Π parameters) that have specific physical interpretations. Prior studies have demonstrated that these Π parameters can be used to provide insight into the biophysical processes involved in expansive growth [23,24,25,26,27,28]. A few Π parameters are important to the analyses conducted here. The subscripts on the Π parameters represent the ratio of specific biophysical processes, e.g., Π_pv_ is interpreted to be the ratio of plastic deformation rate and growth rate in relative volumetric terms, and Π_wv_ is interpreted to be the ratio of the water uptake rate and the growth rate in relative volumetric terms.

$$\Pi_{pv}=\left(\frac{\;\phi \;P_{C}\;}{v_{s}}\;\right)=\left(\frac{\frac{1}{h\;MPa}\frac{MPa}{1}}{\frac{1}{h}}\;\right)=\left(\frac{\;relative\;volumetric\;plastic\;deformation\;rate\;of\;the\;wall}{\;relative\;volumetric\;growth\;rate}\right)$$

$$\Pi_{wv}=\left(\frac{\;L\;P_{C}\;}{\;v_{s}}\;\right)=\left(\frac{\frac{1}{h\;MPa}\frac{MPa}{1}}{\frac{1}{h}}\;\right)=\left(\frac{\;relative\;volumetric\;water\;uptake\;rate}{\;relative\;volumetric\;growth\;rate}\right)$$

A more relevant Π parameter is obtained by dividing Π_pv_ by Π_wv_., which yields the dimensionless number, Π_pw_ [25,28].

$$\Pi_{pw}=\frac{\Pi_{pv}}{\Pi_{wv}}=\left(\frac{\;\phi \;}{L}\right)=\left(\frac{\frac{1}{h\;MPa}}{\frac{1}{h\;MPa}}\;\right)=\left(\frac{\;relative\;volumetric\;plastic\;deformation\;rate\;of\;the\;wall}{\;relative\;volumetric\;water\;uptake\;rate}\right)$$

In Π_pw_, it can be seen that *ϕ* represents the “relative volumetric plastic deformation rate of the wall” and *L* represent the “relative volumetric water uptake rate”.

### 1.4. Method

In this study, the biological passive *P* and *v* responses to changes in water uptake rates are simulated and analyzed for a model (ideal) plant cell. The results of dimensional analyses are used to interpret the physical meaning of relevant biophysical variables in the biophysical equations. Dimensional analyses show that changing the magnitude of the relative hydraulic conductance of the plasma membrane, *L*, can simulate changing the relative volumetric water uptake rate. Here, water deficit is simulated by decreasing the magnitude of *L*. Then, the behavior of *P* and *v* are calculated and analyzed. Because the magnitude of *L* is used to represent the magnitude of “water uptake rate”, the subscripts “w” will be used to remember the “water uptake rate” interpretation; *L*_w_.

It is noted in Equation (2) that the expansive growth rate is related to the magnitude of the plastic deformation rate of the cell wall. And dimensional analyses demonstrate that the magnitude and behavior of *ϕ* can be used to represent the magnitude and behavior of the plastic deformation rate of the wall. Because the magnitude of *ϕ* is used to represent the magnitude of “plastic deformation rate of the wall”, the subscripts “p” will be used to remember the “plastic deformation rate of the wall” interpretation; *ϕ*_p_.

### 1.5. Overview

It is found that *P* and *v* decrease when *L*_w_ decreases in magnitude. Also, it is found that *P* decreases and *v* increases when *ϕ*_p_ increases in magnitude. When *L*_w_ is large (simulating well-watered condition), the decrease in *P* is small and only slightly reduces the increase in *v* caused by the increase in *ϕ*_p_. But when *L*_w_ is small (simulating water deficit condition), the decrease in *P* is large and significantly reduces the increase in *v* caused by the same increase in *ϕ*_p_. This complicated behavior is addressed by incorporating the dimensionless number, $\Pi_{pw}=\left(\frac{\;\phi_{p}\;}{L_{w}}\right)$, into relevant biophysical equations [28]. Then, the magnitude of Π_pw_ increases as the severity of water deficit increases. It is shown that *P* and *v* decrease curvilinearly as Π_pw_ increases. The converted biophysical equations provide another perspective and approach to analyzing experimental results. This perspective and approach are demonstrated with experimental results from studies of maize primary roots growing in water deficit conditions.

## 2. Analyses and Results

### 2.1. Turgor Pressure

The behavior of *P* as a function of time, *P*(*t*), is obtained by solving Equation (3) with the initial condition, *P*(*t* = 0) = *P*_o_ [17].

(4) $$P\left(t\right)={(P}_{o}-P_{\mathrm{eq}})e^{-\;\frac{t}{t_{c}}}+P_{eq}$$

Equation (4) describes the exponential change of *P* from an initial equilibrium constant value, *P*_o_, to another equilibrium constant value, *P*_eq_. The time constant, *t*_c_, for the exponential change is defined by Equation (5).

(5) $$t_{c}=\frac{1}{\varepsilon \;\left(\phi_{p}+L_{w}\right)\;}$$

The magnitude of *P*_eq_ is defined by Equation (6).

(6) $$P_{eq}=\frac{L_{w}\;\Delta \pi +\phi_{p}\;P_{C}}{\phi_{p}+L_{w}}$$

Equations (4)–(6) can be used to study *P*(*t*) when *L*_w_, Δ*π*, *ϕ*_p_, and *P*_C_ change in magnitude individually or in combination.

### 2.2. Turgor Pressure When L_w_ and ϕ_p_ Change

**Figure 1:**
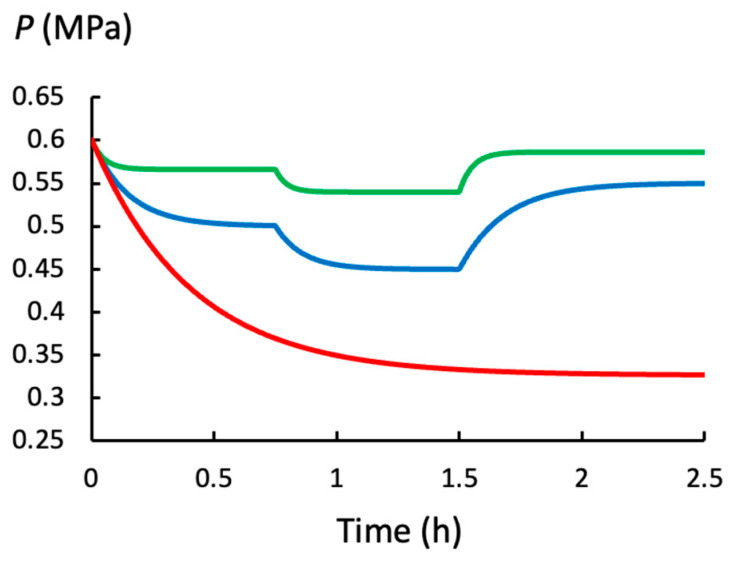
*P*(*t*) for a model (ideal) growing plant cell when well-watered (green curve; *L*_w_ = 2.0 h^−1^ MPa^−1^), during moderate water deficit (blue curve; *L*_w_ = 0.5 h^−1^ MPa^−1^) and during severe water deficit (red curve; *L*_w_ = 0.025 h^−1^ MPa^−1^). The green and blue curves show *P*(*t*) after *ϕ*_p_ increases from 0.0 to 0.25 h^−1^ MPa^−1^ at *t* = 0 h, after *ϕ*_p_ increases from 0.25 h^−1^ MPa^−1^ to 0.5 h^−1^ MPa^−1^ at *t* = 0.75 h, and after *ϕ*_p_ decreases to 0.1 h^−1^ MPa^−1^ at *t* = 1.5 h. In the red curve, *ϕ*_p_ increases 0.0 to 0.25 h^−1^ MPa^−1^ at *t* = 0 h and remains constant for the remainder of the time. The red curve highlights the fact that the transient time between *P*_o_ and *P*_eq_ is much longer during severe water deficits (*L*_w_ = 0.025 h^−1^ MPa^−1^). Table 1 presents the specific values used in Equations (4)–(6) to produce each curve.

The curves for *P*(*t*) in Figure 1 are calculated with Equations (4)–(6) for a model cell that has the biophysical properties similar to those found in growing pea stems, *Pisum sativum* L. [29]; see Table 1. Each *P*(*t*) curve simulates the initiation of elongation growth, at *t* = 0, by making *ϕ*_p_ = 0.25 h^−1^ MPa^−1^ at *t* = 0 h. Also, each *P*(*t*) curve is for a growing cell with a different water uptake rate, i.e., different value of *L*_w_. The top curve (green) represents *P*(*t*) during growth in well-watered conditions (*L*_w_ = 2.0 h^−1^ MPa^−1^), the curve below it (blue) represents *P*(*t*) during growth in moderate water deficit (*L*_w_ = 0.5 h^−1^ MPa^−1^), and the bottom curve (red) represents *P*(*t*) during growth in severe water deficit (*L*_w_ = 0.025 h^−1^ MPa^−1^).

In the top two curves (green and red), the behavior of *P*(*t*) is shown after simulated changes in the wall plastic deformation rate, i.e., after different values of *ϕ*_p_. The value for *ϕ*_p_ increases at *t* = 0 h and *t* = 0.75 h and decreases at *t* = 1.5 h on the time scale. The specific values for all the biophysical variables in each time interval are presented in Table 1.

The following can be observed in Figure 1 and Table 1.

(a) *P*(*t*), *P*_eq_, and *v*_s_ decrease when the rate of water uptake (*L*_w_) decreases.
(b) *P*(*t*) and *P*_eq_ decrease, and *v*_s_ increases, when the wall plastic deformation rate (*ϕ*_p_) increases and *L*_w_ remains constant.
(c) The same increase in *ϕ*_p_ produces a larger decrease in *P*(*t*) and *P*_eq_ when *L*_w_ is smaller, and this produces a smaller increase in *v*_s_.
(d) When *L*_w_ and/or *ϕ*_p_ decrease, *t*_c_ increases. In general, changes in *P*(*t*) and *v*(*t*) take longer to complete when *L*_w_ is small.

It is noted that the quantitative information in *P*(*t*) is contained in its two components, *P*_eq_ and *t*_c_, see Equations (4)–(6). Therefore, quantitative analyses of *P*(*t*) are performed by conducting quantitative analyses of *P*_eq_ and *t*_c_.

### 2.3. Analyses of the Time Constant, t_c_

The *t*_c_ for each curve is calculated using Equation (5) and presented for each time interval in Table 1. Note that the magnitude of *t*_c_ increases when the term, *ε* (*ϕ*_p_ + *L*_w_), decreases in magnitude. And the time required for transition between different magnitudes of *P*_eq_ increases when *t*_c_ increases. For an exponential change, most of the transition is completed in approximately 4*t*_c_. So, time of transition between different magnitudes of *P*_eq_ can be estimated as 4*t*_c_. In general, *t*_c_ and the transition time increase during water deficit because *L*_w_ is always smaller compared to well-watered conditions. Interestingly, plant cells with stiffer walls (larger values for *ε*) have faster transition times than cells with smaller values for *ε*. Generally, more mature walls are stiffer than those of growing plant cells.

### 2.4. Analyses of the Equilibrium Turgor Pressure, P_eq_

Equation (6) describes *P*_eq_. It is noted that *P*_eq_ is a function of *ϕ*_p_, *L*_w_, Δ*π*, and *P*_C_. Examination of Equation (6) shows that the relationship between *P*_eq_, Δ*π*, and *P*_C_ is straight-forward, i.e., *P*_eq_ increases and decreases when Δ*π* and *P*_C_ increase and decrease individually or in combination. However, the relationship between *P*_eq_, *ϕ*_p_, and *L*_w_ is more complicated. Graphing *P*_eq_ as a function of *ϕ*_p_ and *L*_w_ requires multiple curves, where one variable (*L*_w_ or *ϕ*_p_) is held constant while the second variable changes. This is similar to what was performed in Figure 1 where *L*_w_ is constant for each curve and *ϕ*_p_ is changed (green and blue curves). Importantly, employing a dimensionless number, $\Pi_{pw}=\left(\frac{\;\phi \;}{L}\right)$, allows Equation (6) to be modified so that a single curve can describe *P*_eq_ when *L*_w_ and *ϕ*_p_ change, individually or concurrently [28]. Notice that Π_pw_ increases when *ϕ* increases and *L* decreases. Equation (7) is a modified expression for *P*_eq_ that incorporates $\Pi_{pw}=\left(\frac{\;\phi_{p}\;}{L_{w}}\right)$, (see Appendix B for details).

(7) $$P_{eq}=\frac{\;\Delta \pi +\Pi_{pw}\;P_{C}}{1+\Pi_{pw}}$$

**Figure 2:**
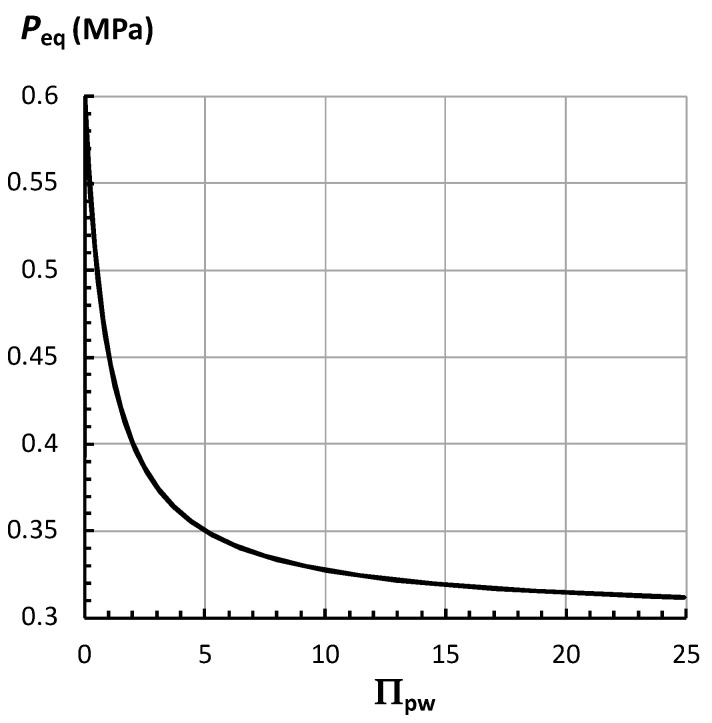
Equation (7) is used to plot the equilibrium of turgor pressure, *P*_eq_, as a function of $\Pi_{pw}=\left(\frac{\;\phi_{p}\;}{L_{w}}\right)$. The values for Δ*π* and *P*_C_ are 0.6 MPa and 0.3 MPa, respectively.

In Equation (7), when Δπ and *P*_C_ are constant, *P*_eq_ is only a function of Π_pw_. Therefore, *P*_eq_ can be plotted by a single curve. Figure 2 shows that as Π_pw_ increases, *P*_eq_ decreases curvilinearly. The decrease in *P*_eq_ is large when Π_pw_ increases from zero to five. The decrease is more gradual for larger values of Π_pw_. It is noted that *P*_eq_ approaches *P*_C_ (*P*_C_ = 3.0 MPa) as Π_pw_ increases but never becomes *P*_C_.

A dimensionless equilibrium turgor pressure, *P*_eq_*, as a function of Π_pw_ can be obtained; Equation (8) (see Appendix C for details).

(8) $$P_{eq}^{*}=\frac{P_{eq}-P_{C}}{\Delta \pi -P_{C}}=\frac{1}{1+\Pi_{pw}}$$

**Figure 3:**
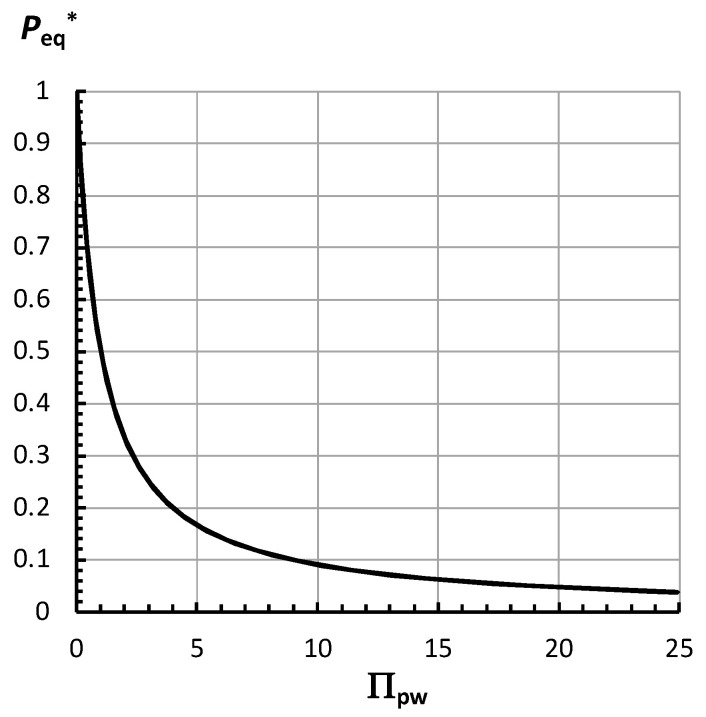
Equation (8) is used to plot the dimensionless equilibrium turgor pressure, *P*_eq_*, as a function of $\Pi_{pw}=\left(\frac{\;\phi_{p}\;}{L_{w}}\right)$.

Figure 3 is a plot of *P*_eq_* versus Π_pw_.

It can be seen that Figure 3 is similar to Figure 2 and that *P*_eq_* decreases curvilinearly as Π_pw_ increases. Equation (8) shows that when Π_pw_ increases to very large magnitudes, *P*_eq_* becomes very small and approaches zero, but never becomes zero. Note that *P*_eq_ can be obtained for any value of Π_pw_ from Equation (8), as well as from Equation (7), (see Appendix D for an example).

### 2.5. Growth Rate for Gradual Changes in P

The relative rate of deformation of the cell wall chamber, *v*_cw_, is the sum of the plastic and elastic relative rates of deformation; Equation (2). Because expansive growth is defined as the permanent increase in the size (volume) of a walled cell, only the rate of plastic deformation rate contributes to the expansive growth rate. Equation (9) describes the relative expansive growth rate of a walled cell as a function of time, *v*(*t*).

(9) $$v\left(t\right)=\phi_{p}\;\left[P\left(t\right)-P_{C}\right]$$

**Figure 4:**
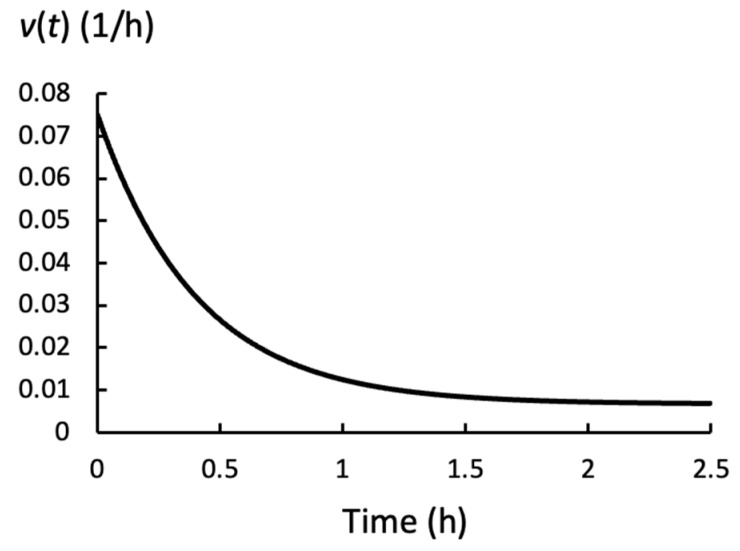
The time-dependent relative growth rate, *v*(*t*), calculated using Equation (9) and *P*(*t*) for the red curve in Figure 1.

Equation (9) ignores the elastic deformation rate and cannot account for fast changes in *P*(*t*). In those cases, Equation (2) more accurately determines the relative rate of deformation of the cell wall chamber. However, Equation (9) is relatively accurate for the slow changes in *P*(*t*) that are observed during water deficits, similar to those shown by the red curve in Figure 1. Figure 4 shows the time-dependent relative growth rate, *v*(*t*), that is calculated using Equation (9) and the *P*(*t*) for the red curve in Figure 1.

### 2.6. Steady Relative Growth Rate, v_s_, i.e., When P = Constant

During the equilibrium periods, *P* = *P*_eq_ = constant and d*P*/d*t* = 0. Then, Equation (10) can be used to determine the steady relative growth rate, *v*_s_ [15,16,17]. The *P*_eq_ used in Equation (10) can be determined with either Equations (6)–(8).

(10) $$v_{s}=\phi_{p}\;\left(P_{eq}-P_{C}\right)$$

Also, when *P* is constant, Equation (11) can be used to determine the steady relative growth rate, *v*_s_ [16]. Equation (11) is obtained by substituting Equation (6) into Equation (10).

(11) $$v_{s}=\frac{\phi_{p}\;L_{w}}{\phi_{p}+L_{w}}\;\left(∆\pi -P_{C}\right)$$

Equation (11) has the advantage of eliminating *P*_eq_ from Equation (10) and making the dependence of *v*_s_ on *ϕ*_p_ and *L*_w_ explicit. This advantage is balanced by a disadvantage that the relationship between *v*_s_, *ϕ*_p_, and *L*_w_ is more complicated and difficult to visualize, graph, and understand [28]. An equation that is simpler to graph and understand is Equation (12). Equation (12) is obtained by incorporating $\Pi_{pw}=\left(\frac{\;\phi_{p}\;}{L_{w}}\right)$ into Equation (11) [28]; see Appendix E for details.

(12) $$v_{s}=\frac{1}{1+\Pi_{pw}}\;\phi_{p}\;\left(∆\pi -P_{C}\right)$$

For convenience in subsequent analyses, we will define, *ϕ*_p_ (Δ*π* − *P_C_*), as *v*_s(max)_.

(13) $$v_{s\left(max\right)}=\phi_{p}\;\left(∆\pi -P_{C}\right)$$

where *v*_s(max)_ is an unachievable theoretically maximum steady growth rate for any magnitude of *ϕ*_p_ [28]. Notice that the term, 1/(1 + Π_pw_), represents a fractional coefficient of *v*_s(max)_. The fractional coefficient, 1/(1 + Π_pw_), is identical to the expression for *P*_eq_* in Equation (8). So, the curve in Figure 3 is a plot of both *P*_eq_* and the fractional coefficient, 1/(1 + Π_pw_), versus Π_pw_. Therefore, we can write Equation (14) [28].

(14) $$v_{s}=P_{eq}^{*}\;v_{s\left(max\right)}$$

**Figure 5:**
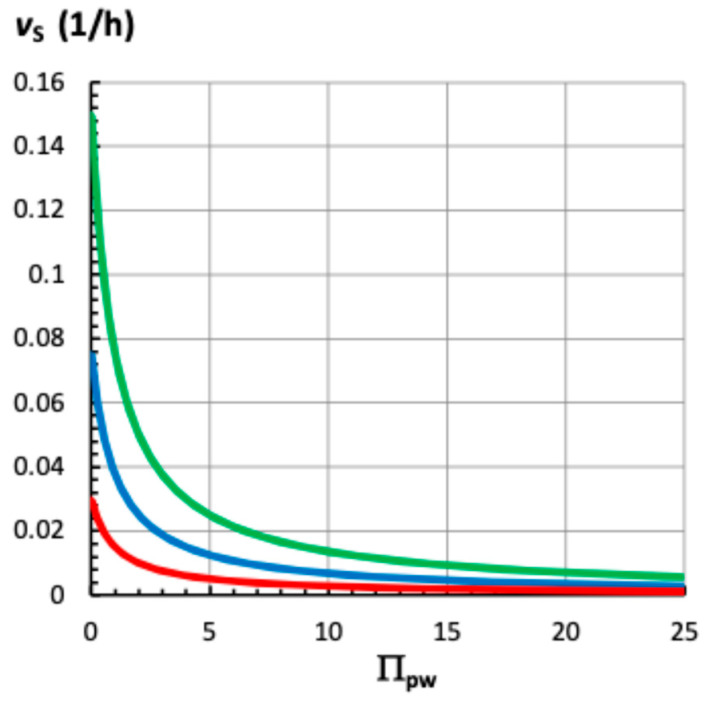
The steady relative growth rate, *v*_s_, versus $\Pi_{pw}=\left(\frac{\;\phi_{p}\;}{L_{w}}\right)$ when *ϕ*_p_ = 0.50 h^−1^ MPa^−1^ (green curve), *ϕ*_p_ = 0.25 h^−1^ MPa^−1^ (blue curve), and *ϕ*_p_ = 0.10 h^−1^ MPa^−1^ (red curve). Equation (12) was used to calculate *v*_s_ as a function of Π_pw_. The values for Δ*π* and *P*_C_ were the same for all curves; Δ*π* = 0.6 MPa and *P*_C_ = 0.3 MPa.

Now *v*_s_ is easily calculated by using *P*_eq_*. As an example, when $\Pi_{pw}=\left(\frac{\;\phi_{p}\;}{L_{w}}\right)=2$, Equation (8) is used to determine that *P*_eq_* = 1/3. Using Equation (14), *v*_s_ is calculated to be 1/3 *v*_s(max)_, i.e., *v*_s_ = 0.333 *v*_s(max)_. Equation (13) shows that *v*_s(max)_ is a function of *ϕ*_p_. Figure 5 shows *v*_s_ plotted against Π_pw_ for three different values of *ϕ*_p_.

It can be seen that *v*_s_ behaves similarly for all magnitudes of *ϕ*_p_, a large decrease in *v*_s_ occurs when Π_pw_ increases from zero to five. For larger Π_pw_, the decrease in *v*_s_ is smaller.

## 3. Discussion

### 3.1. Turgor Pressure During Water Deficits

The behavior of the time-dependent turgor pressure, *P*(*t*), of a plant cell during water deficits is simulated by decreasing the magnitude of *L*_w_ (relative volumetric water uptake rate) and calculating *P*(*t*) with Equation (4). Figure 1 shows that *P*(*t*) decreases exponentially to a smaller magnitude when *L*_w_ decreases in magnitude. This simple behavior is complicated by the fact that an increase in *ϕ*_p_ (relative volumetric plastic deformation rate of the wall) increases the growth rate but also decreases *P*(*t*). In well-watered cells, i.e., when *L*_w_ is large, the decrease in *P*(*t*) is small. But for cells growing in water deficit, i.e., when *L*_w_ is small, the same increase in *ϕ*_p_ produces a larger decrease in *P*(*t*), see Figure 1 and Table 1. This finding demonstrates that *ϕ*_p_ must be part of the analyses of *P*(*t*) during water deficits.

A quantitative analysis of the relationship between *P*(*t*), *L*_w_, and *ϕ*_p_ was conducted with analyses of the components of *P*(*t*); *P*_eq_ (equilibrium turgor pressure) and *t*_c_ (time constant). The *t*_c_ was calculated using Equation (5). The magnitudes of *t*_c_ for the curves shown in Figure 1 are presented in Table 1. It can be seen that *t*_c_ increases when *L*_w_, *ϕ*_p_, and *ε* decreases in magnitude, individually or in combination. So, during water deficits when *L*_w_ is smaller, the *t*_c_ is always larger and changes in *P*(*t*) are always slower.

Equation (6) shows that the relationship between *P*_eq_, *L*_w_, and *ϕ*_p_ is complicated and difficult to interpret. Employing the dimensionless number, $\Pi_{pw}=\left(\frac{\;\phi_{p}\;}{L_{w}}\right)$, simplifies the relationship and shows that *P*_eq_ depends on Π_pw_; Equation (7). It was noted that the severity of water deficit increased as the magnitude of Π_pw_ increased in magnitude. This simplification allows *P*_eq_ to be plotted against Π_pw_; Figure 2. A dimensionless *P*_eq_ (*P*_eq_*) was derived, Equation (8), and plotted against Π_pw_; Figure 3. In both Figure 2 and Figure 3, it is shown that *P*_eq_ and *P*_eq_* decrease curvilinearly as Π_pw_ increases. The largest decrease occurs when Π_pw_ increases from zero to five. It was noted that *P*_eq_ approached *P*_C_ at very large values of Π_pw_, but never became *P*_C_. And *P*_eq_* approached zero at very large values of Π_pw_, but never became zero.

### 3.2. Expansive Growth During Water Deficit

Table 1 shows that the steady relative growth rate, *v*_s_, decreases when *L*_w_ decreases. And it is shown that *v*_s_ increases after an increase in *ϕ*_p_, but the magnitude of the increase in *v*_s_ is smaller for the same increase in *ϕ*_p_ when *L*_w_ is smaller. This occurs because *P*(*t*) decreases after an increase in *ϕ*_p_, and the decrease is larger when *L*_w_ is smaller. During water deficits the behavior of *v*(*t*) is predominately determined by *P*(*t*), and because the *t*_c_ is large, the changes in *P*(*t*) are slow. Therefore, Equation (9) can provide an accurate approximation of *v*(*t*) when *L*_w_ is small; see Figure 4.

The *v*_s_ during well-watered and water deficit conditions is determined by Equation (10) that shows *v*_s_ depends on *P*_eq_. And *P*_eq_ depends on both *ϕ*_p_ and *L*_w_, see Equation (6). Thus, *v*_s_ implicitly depends on *ϕ*_p_ and *L*_w_. This dependence can be made explicit, but doing so complicates the relationship and interpretation, see Equation (11). As in the case with *P*_eq_, employing $\Pi_{pw}=\left(\frac{\;\phi_{p}\;}{L_{w}}\right)$ simplifies the relationship, e.g., Equations (12)–(14), and allows the relationship between *v*_s_ and Π_pw_ to be plotted on a single graph for different values of *ϕ*_p_; Figure 5. All the curves in Figure 5 are similar, showing a large decrease in *v*_s_ when Π_pw_ increases from zero to five, and a smaller decrease when Π_pw_ is greater than five. This behavior is similar to that of *P*_eq_ and *P*_eq_* versus Π_pw_. It can be seen that larger magnitudes of *ϕ*_p_ shift the curves upward so that larger growth rates occur at the same magnitude of Π_pw_.

### 3.3. Analyzing Expansive Growth During Water Deficits

Employing Π_pw_ to understand changes of *P*_eq_ and *v*_s_ to water deficits provides another perspective and approach when analyzing experimental results. This approach consists of determining or estimating the magnitude of Π_pw_ after a change in water conditions, and then employing Figure 2, Figure 3 and Figure 5 to determine how much *P*_eq_ and *v*_s_ change. It is simple to think that *P*_eq_ and *v*_s_ decrease as Π_pw_ increases. Furthermore, determining a magnitude for Π_pw_ provides a simple method for obtaining a quantitative interpretation of how much *P*_eq_ and *v*_s_ change as the severity of water deficits increases; Equations (7), (8), (12), and (14).

Equations (12) and (14) are simple equations that can be used to analyze experimental results. Recognizing that the curves shown in Figure 5 are calculated when *v*_s(max)_ = constant (a different constant for each value of *ϕ*_p_), then each curve represents the “passive response” of *v*_s_ to water deficit. Each curve can be thought of as being composed of two parts, *P*_eq_* and *v*_s(max)_. The behavior of *P*_eq_* as a function of Π_pw_ is described by Equation (8) and this relationship remains the same. However, the behavior of *v*_s(max)_ is described by Equation (13) and does not have to remain constant, i.e., *ϕ*, Δ*π*, and *P_C_* may change in magnitude (adjust) as water deficits become more severe. Therefore, any deviation of *v*_s_ from the “*v*_s_ versus Π_pw_” curve (when the values are constant), will indicate an “active response” during water deficit. Inspection of Equation (13) demonstrates that an increase in the magnitude of *ϕ* and/or (Δ*π* − *P_C_*) will increase *v*_s(max)_ and will therefore shift the “*v*_s_ versus Π_pw_” curve upward, see Figure 5. Of course, a decrease in the magnitude of *ϕ* and/or (Δ*π* − *P_C_*) decreases *v*_s(max)_ and will shift the “*v*_s_ versus Π_pw_” curve downward so that smaller growth rates occur at the same magnitude of Π_pw_.

The sensitivity of *v*_s_ to active changes in the magnitude of *ϕ*_p_, Δ*π*, and *P_C_* can be calculated using Equation (14). The magnitude of *P*_eq_* for different values of Π_pw_ will always be the same, so the magnitude of the change in *v*_s(max)_ will determine the magnitude of the change in *v*_s_. In other words, the % change (positive or negative) of *v*_s_ will equal the % change (positive or negative) of *v*_s(max)_ for different values of Π_pw_ and for different ranges of Π_pw_. For example, when Π_pw_ = 5, then *P*_eq_* = 1/6, see Equation (8). Using *ϕ*_p_ = 0.25 h^−1^ MPa^−1^, Δ*π* = 0.6 MPa, and *P_C_* = 0.3 MPa, then *v*_s(max)_ = 0.075 h^−1^ is calculated, see Equation (13). Equation (14) is used to calculate *v*_s_ = 0.0125 h^−1^. Now if *ϕ*_p_ increases two-fold (100%) to 0.5 h^−1^ MPa^−1^, then *v*_s(max)_ = 0.15 h^−1^ and *v*_s_ = 0.025 h^−1^, which represent a two-fold (100%) increase in relative growth rate. It is apparent that the magnitude of the change in *v*_s(max)_ determines the magnitude of the change in *v*_s_ for a specific value of Π_pw_. When *ϕ*_p_, Δ*π*, and *P_C_* change individually or in combination, Equation (13) can be used to determine the percent increase or decrease in *v*_s(max)_, which represents the percent increase or decrease in *v*_s_ for different individual values, or range of values, of Π_pw_. It follows that if *ϕ*_p_ increases from 0.25 h^−1^ MPa^−1^ to 0.5 h^−1^ MPa^−1^ beginning at Π_pw_ = 5 and extending to Π_pw_ = 25, the blue curve in Figure 5 will be shifted to the position of the green curve for that range of Π_pw_, i.e., 5 ≤ Π_pw_ ≤ 25.

The sensitivity of *P*_eq_ to active changes in the magnitude of Δ*π* and *P_C_* is more complicated to determine, but the magnitude of the changes can be calculated using Equation (7). As an example, if Δ*π* increases from 0.6 MPa to 0.9 MPa (50% increase) and *P_C_* = 0.3 MPa, then Equation (7) can be used to calculate *P*_eq_ = 0.4 MPa when Π_pw_ = 5. An inspection of Figure 2 indicates that *P*_eq_ = 0.35 MPa when Δ*π* = 0.6 MPa, *P_C_* = 0.3 MPa, and Π_pw_ = 5. So, *P*_eq_ has increased 0.05 MPa (14.3%) when Δ*π* increased 0.3 MPa (50%) at Π_pw_ = 5. However, when Π_pw_ = 15, the same increase in Δ*π* of 0.3 MPa (50%) will increase *P*_eq_ from 0.32 MPa to 0.34 MPa, which represents an increase of 0.02 MPa (6%). Summarizing, a 50% increase in Δ*π* produces an increase in *P*_eq_ of 14.3% at Π_pw_ = 5, and 6% at Π_pw_ = 15. This example reveals that the sensitivity of *P*_eq_ on Δ*π* depends on the magnitude of Π_pw_. This occurs because Π_pw_ is present in both the numerator and denominator of Equation (7).

Theoretically, it is not possible to determine the value(s) of Π_pw_ that will elicit an active response. It seems reasonable that the large decreases in *P*_eq_ and *v*_s_ that occur between Π_pw_ = 0 and Π_pw_ = 5 would elicit an active response. However, only analyses conducted on experimental results can indicate where “*v*_s_ versus Π_pw_” curves deviate from passive response curves to indicate an active response

### 3.4. Growth Rate and Turgor Pressure of Roots During Water Deficits

Remembering that the analyses conducted here do not consider the transpiration rate from single plant cells. Because roots do not transpire to the atmosphere or to the soil [30], the expansive growth of roots in water deficits can be evaluated with these biophysical equations and analyses. In order to extend these analyses from growing plant cells to growing plant tissues and organs, a “lump” parameter method is employed. This method considers the behavior of a group of cells (lumped together) and the lump is analyzed as a single cell. The main advantage of this approach is that simpler equations can be used for analyses (those of a single cell) and the “system” behavior of the tissue and/or organ can be assessed. Care should be exercise in these analyses, because the magnitudes of relevant variables and parameters in the equation(s) are an average of all the cells in the lump. In general, information is always lost when quantities are averaged. So important behavior of some cells within the lump that are different can be lost in the average. However, the “system” behavior of the lump of cells can be useful and can provide insight that may be difficult or impossible to obtain by focusing on individual cells. Generally, this method yields the best results when the cells in the lump are homogenous in structure, function, and behavior—such as cells in growing tissue. The lump parameter approach has been implicitly employed in prior investigations [29,30,31] and have revealed important system behavior and insights.

### 3.5. Growth Rate and Turgor Pressure of Maize Roots During Water Deficits

Sharp et al. [32] grew seedlings of maize (*Zea mays* L.) in vermiculite at decreasing water potentials, *Ψ*_w_. The steady growth rates of primary roots were measured and plotted as a function of the decreasing *Ψ*_w_ of the vermiculite. The curve in Figure 1 of their paper [32] is similar to the curves in Figure 5 presented here.

#### 3.5.1. Passive Response

By considering the growth zone of the primary root as a lumped parameter (from the apex to approximately 10 mm from the apex) the analyses conducted here can be extended to evaluate the results presented in Figure 1 [32]. Two features of Figure 1 [32] were noted and compared to the curves in Figure 5. One feature is that a large decrease in root elongation rate (growth rate) occurs when *Ψ*_w_ decreases from −0.03 MPa to −0.5 MPa (this is similar to the behavior of *v*_s_ when Π_pw_ decreases from 0 to 5 in Figure 5). The second feature is that the decrease in growth rate is smaller when *Ψ*_w_ decreases from −0.5 MPa to −2.0 MPa (this is similar to the behavior of *v*_s_ when Π_pw_ decreases from 5 to 25 in Figure 5).

Employing the analyses and equations presented here, a prediction can be made. It is predicted that a decrease in turgor pressure is primarily responsible for the decrease in growth rate when *Ψ*_w_ decreases from −0.03 MPa to −2.0 MPa. This prediction is made because Equation (14) and Figure 2, Figure 3, Figure 4 and Figure 5 demonstrate that the overall shape of the “growth rate versus water deficit” curve is determined primarily by the behavior of the turgor pressure as a function of increasing water deficit. A subsequent investigation by Spollen and Sharp [33], working with maize primary roots, provides partial confirmation of this prediction. In that investigation, a pressure probe was used to measure the turgor pressure in individual cells located in the apical 10 mm length of the primary root. It was shown that the turgor pressure was nearly uniform and constant along the 10 mm length. Furthermore, the turgor pressure was smaller when *Ψ*_w_ of the vermiculite was smaller. In their Figure 2 [33], it is shown that the approximate average turgor pressure was, *P*_ave_ = 0.7 MPa when *Ψ*_w_ = −0.02 MPa, and *P*_ave_ = 0.3 MPa when *Ψ*_w_ = −1.6 MPa. These results are consistent with the behavior of *P*_eq_ and *P*_eq_* as a function of Π_pw_ that are shown in Figure 2 and Figure 3.

#### 3.5.2. Active Response

A careful comparison of Figure 1 in Sharp et al. [32] and Figure 5 indicates that an upward shift in the “root elongation rate versus vermiculite water potential” curve occurs approximately between −0.3 MPa and −2.0 MPa. This upward shift corresponds to the approximate range of 3 < Π_pw_ < 25 of the “*v*_s_ versus Π_pw_” curve in Figure 5. The upward shift indicates an active response, and that *v*_s(max)_ has increased in magnitude. Inspection of Equation (13) demonstrates that an increase in the magnitude of *ϕ* and/or (Δ*π* − *P_C_*) increases *v*_s(max)_ and shifts the “*v*_s_ versus Π_pw_” curve upward, see Figure 5. Then, it is predicted that *ϕ* and/or Δ*π* must have increased in magnitude, or that *P*_C_ decreased in magnitude when *Ψ*_w_ was between −0.3 MPa and −2.0 MPa.

In another investigation by Sharp et al. [34], also working with maize primary roots, it was shown that there was substantial osmotic adjustment in the growth zone of the primary root of maize at low *Ψ*_w_. Interestingly, the osmotic adjustment was largely the result of a greater inhibition of volume expansion and water deposition than solute deposition. An increase in Δ*π* in the range of 3 < Π_pw_ < 25 in Figure 5 will shift the curves upward in that range. Inspection of Equation (7) demonstrates that *P*_eq_ will also be shifted upward in the same range of 3 < Π_pw_ < 25 of Figure 2.

#### 3.5.3. Limitation of the “Lump” Parameter Method

Sharp et al. [32] employed time lapse photography to reveal that elongation growth near the root apex was insensitive to water potentials as low as −1.6 MPa. Instead, growth was inhibited in more basal locations, decreasing the growth zone length progressively as the water potential decreased. These findings highlight a limitation of the “lump” parameter method, because only the total elongation rate of the root is analyzed in this method. So, the distribution of growth along the length of the root and the decrease in growth zone length go undetected in the “lump” parameter method. Other changes within the lumped volume of cells are neglected, some of which provide important insight into the growth process. In general, it is useful to supplement the “lump” parameter findings with more in-depth studies.

### 3.6. Growth Rate of Roots and Shoots from Other Plants During Water Deficits

The analyses, equations, and results presented here draw support from other species of plants. The curves of elongation rates of the primary root and shoot of seedlings from maize, soybean, cotton, and squash at decreasing water potentials are similar to those in Figure 5 [35,36]. The shoots were grown in saturating humidity in an attempt to suppress transpiration. Both roots and shoots show a large curvilinear decrease in elongation rate when *Ψ*_w_ decreases from 0.0 MPa to −0.5 MPa, with the shoots showing the largest decrease; see Figure 3.1 in [35] and Figure 1 in [36]. For the roots, the curves demonstrate a more gradual curvilinear decrease in elongation rate when *Ψ*_w_ decreases from 0.0 MPa to −1.5 MPa. The results demonstrate that the roots of these plants continue to grow at water potentials that are low enough to completely inhibit shoot growth. The differences in the curves of “elongation rate versus *Ψ*_w_” for maize, soybean, cotton, and squash roots [35,36] indicate a different magnitude of *v*_s(max)_ in Equation (13). In general, experimental research has shown that each of the variables in Equation (13) have been actively altered during water deficit [6,32,33,34,35,36,37].

### 3.7. Transpiration and Apoplasm Pathway

Because the theoretical analyses conducted here neglect the transpiration water loss from individual cells, the turgor pressure and growth rate behavior presented in Figure 1, Figure 2, Figure 3, Figure 4 and Figure 5 are strictly only for plant cells that are not transpiring. However, in addition to growing roots, the results of these analyses might be extended to shoots, stems, and leaves growing in a high humidity environment that suppresses transpiration, e.g., in the laboratory or in the tropics. Future research will focus on incorporating “transpiration” into analyses similar to that conducted here. However, the analyses are complicated by the fact that transpiration from exterior cells lowers the pressure in the apoplasm pathway (cell walls and xylem), which in turn lowers the turgor pressure of the cells throughout the growing plant tissue or organ [20]. Previously, the biophysical equations were modified to evaluate the turgor pressure, water uptake rate, and expansive growth rate of plant cells in tissue when pressures within the apoplasm were lower and higher than atmospheric pressure [20]. The obtained governing equations are more complicated than those presented here. Therefore, the objective of future research is to simplify the analyses and equations, so they are similar to those presented here, and perhaps easier to understand and use than those previously obtained [20].

## 4. Conclusions

Here, equations derived from previously validated biophysical equations describe the behavior of the turgor pressure and expansive growth rate of plant cells as water deficit increases in severity; see Equations (7) and (8) and Equations (12)–(14), respectively. The new equations employ a dimensionless number, $\Pi_{pw}=\left(\frac{\;\phi_{p}\;}{L_{w}}\right)$, that increases in magnitude as the severity of water deficit increases. The equations neglect transpiration water losses, so their applications are limited to plant cells that are not transpiring, i.e., roots, and non-transpiring shoots, stems, and leaves growing in saturating high humidity. The steady relative growth rate, *v*_s_, is described by Equation (14) and simply consists of the product of two variables, *P*_eq_* and *v*_s(max)_. Each variable is represented by an equation, Equations (8) and (13), both of which are simple and easy to understand. Figure 5 shows that *v*_s_ decreases curvilinearly as the water deficit severity (Π_pw_) increases. The curvilinear behavior of *v*_s_ is described by the dimensionless equilibrium turgor pressure, *P*_eq_*, which was derived from the equilibrium turgor pressure, *P*_eq_, Equation (7). The effect of changes in the magnitudes of *ϕ*_p_, Δ*π*, and *P_C_* on *v*_s_ at different Π_pw_, or ranges of Π_pw_, can be determined by calculating the magnitude of *v*_s(max)_ with Equation (13) and using Equation (14) to calculate *v*_s_. Adjusting the magnitude of *v*_s(max)_ can fit the curves shown in Figure 5 to those of different species of plants. Also, adjusting the magnitude of *v*_s(max)_ for a range of Π_pw_ can determine the magnitude of an active response during water deficit.

The growth rate behavior presented in Figure 5 is similar to experimental results of roots and shoots (shoots that were grown in saturating high humidity) from four different species of plants grown in increasing water deficit [35,36]. The large decrease in *P*_eq_ as Π_pw_ increases, that is shown in Figure 2, is consistent with experimental results from pressure probe studies of maize roots grown in water deficit [33]. Therefore, it is concluded that the equations derived here can describe the behavior of the turgor pressure and expansive growth rate of non-transpiring plant cells as water deficits increase in severity.

The derived equations and findings can have real-world applications. For example, Figure 5 shows that you obtain the best return on your investment if you apply growth stimulants to well-watered crops (small Π_pw_) compared to crops in water deficit (larger Π_pw_). A growth stimulant that produces a 100% increase in *v*_s_, will increase the relative growth rate from 0.06/h to 0.12/h when Π_pw_ < 1.0 (see Figure 5, blue curve to green curve), but will only increase the growth rate from 0.013/h to 0.025/h when Π_pw_ = 5.0. Also, the finding that *v*_s_ approaches zero asymptotically as Π_pw_ increases, but never becomes zero (Figure 5), may find applications in irrigation management and ecology. It would appear that the most effective use of limited amounts of water to maintain plant growth in dry climates is to frequently distribute small quantities of water over a period of time, compared to distributing larger quantities of water less frequently over the same time period. Also, the derived equations may provide guidance for drought-tolerant crop breeding.

## Figures and Tables

**Table 1:** The numerical values for the curves in Figure 1 are presented here. The values in the first four rows (for Δ*π*, *P*_C_, *ε*, and *ϕ*_p_) are common to all curves, except for the last two time-intervals of the red curve. The values in the next five rows (for *L*_w_, *P*_eq_, *t*_c_, Π_pw_, and *v*_s_) are for the green curve, and the next five rows are for the blue curve. The last five rows are for the red curve and for a single a time interval of 2.5 h. The steady relative growth rate, *v*_s_, was calculated with Equation (10).

Biophysical Variable (Units)	*t* < 0	Time Intervals 0.0 h ≤ *t <* 0.75 h	0.75 h ≤ *t <* 1.5 h	1.5 h ≤ *t <* 2.5 h
Δ*π* (MPa)	0.6	0.6	0.6	0.6
*P*_C_ (MPa)	---	0.3	0.3	0.3
*ε* (MPa)	9.0	9.0	9.0	9.0
*ϕ*_p_ (h^−1^ MPa^−1^)	0.0	0.25	0.50	0.10
		**For Green Curve**		
*L*_w_ (h^−1^ MPa^−1^)	2.0	2.0	2.0	2.0
*P*_eq_ (MPa)	0.60	0.566	0.540	0.586
*t*_c_ = (h)	---	0.049	0.044	0.053
Π_pw_	---	0.125	0.25	0.05
*v*_s_ (h^−1^)	---	0.067	0.12	0.029
		**For Blue Curve**		
*L*_w_ (h^−1^ MPa^−1^)	2.0	0.5	0.5	0.5
*P*_eq_ (MPa)	0.60	0.500	0.450	0.550
*t*_c_ = (h)	---	0.148	0.111	0.185
Π_pw_	---	0.5	1.0	0.2
*v*_s_ (h^−1^)	---	0.05	0.075	0.025
		**For Red Curve**		
	***t *** **< 0**	**0.0 h ≤ ** ***t <*** ** 2.5 h**		
*L*_w_ (h^−1^ MPa^−1^)	2.0	0.025		
*P*_eq_ (MPa)	0.60	0.327		
*t*_c_ = (h)	---	0.40		
Π_pw_	---	10.0		
*v*_s_ (h^−1^)	---	0.00675		

## Data Availability

The original contributions presented in this study are included in the article. Further inquiries can be directed to the corresponding author.

## Appendix A. Definitions of Individual Variables (Units)

*A*—area of the plasma membrane (m^2^)
*L*_p_—hydraulic conductivity of the plasma membrane (m h^−1^ MPa^−1^)
*L*—relative hydraulic conductance of the plasma membrane $=\left(\frac{L_{p}A}{\;V}\right)$ (h^−1^ MPa^−1^)
*P*—turgor pressure (MPa)
*t*—time (h)
*V*—volume (m^3^)
*v*—relative rate of change in volume (h^−1^)
*ε*—volumetric elastic modulus of the cell wall (MPa)
*ϕ*—relative irreversible extensibility of the cell wall (h^−1^ MPa^−1^)
*π*—osmotic pressure (MPa)
Δ*π*—difference in osmotic pressure across the plasma membrane (MPa)
Π—dimensionless parameter or dimensionless number (no units)

## Appendix B. Derivation of Equation (7)

Beginning with the expression, $\Pi_{pw}=\left(\frac{\;\phi_{p}\;}{L_{w}}\right)$, then $\phi_{p}=\;L_{w}\Pi_{pw}$.

Now substituting into Equation (6) we get the expression for Equation (7).

$$P_{eq}=\frac{L_{w}\;\Delta \pi \;+\;\phi_{p}\;P_{C}}{\phi_{p}+L_{w}}=\frac{L_{w}\;\Delta \pi \;+\;L_{w}\;\Pi_{pw}\;P_{C}}{L_{w}\;\Pi_{pw}+\;L_{w}}=\frac{L_{w}\;\left(\Delta \pi \;+\;\Pi_{pw}\;P_{C}\right)}{L_{w}\;\left(\Pi_{pw}+\;1\right)}=\frac{\;\Delta \pi \;+\;\Pi_{pw}\;P_{C}}{\;\Pi_{pw}+\;1}$$

## Appendix C. Derivation of Equation (8)

Begin by substituting Equation (7) into the expression for *P*_eq_*, i.e., $P_{eq}^{*}=\frac{P_{eq}-P_{C}}{\Delta \pi -P_{C}}$. Then, the expression for Equation (8) is obtained.

$$P_{eq}^{*}=\frac{P_{eq}\;-\;P_{C}}{\Delta \pi \;-\;P_{C}}=\frac{\;\left(\frac{\;\Delta \pi +\Pi_{pw}\;P_{C}}{1+\Pi_{pw}}\right)\;-\;P_{C}}{\Delta \pi \;-\;P_{C}}=\frac{\;\left(\frac{\;\Delta \pi \;+\;\Pi_{pw}\;P_{C}}{1+\Pi_{pw}}\right)-P_{C}\;\left(\frac{\;1\;+\;\Pi_{pw}\;}{1\;+\Pi_{pw}}\right)}{\Delta \pi \;-\;P_{C}}$$

$$=\frac{\;\left(\frac{\Delta \pi \;+\;\Pi_{pw}\;P_{C}\;-\;P_{C}-\Pi_{pw}\;P_{C}}{1+\Pi_{pw}}\right)\;}{\Delta \pi \;-\;P_{C}}=\frac{\;\left(\frac{\;\Delta \pi \;-P_{C}}{1+\Pi_{pw}}\right)\;}{\Delta \pi \;-\;P_{C}}=\frac{\left(\Delta \pi \;-P_{C}\right)}{\left(\Delta \pi \;-\;P_{C}\right)\;\left(1+\Pi_{pw}\right)}=\frac{1}{1+\Pi_{pw}}$$

## Appendix D. Using Equation (8) to Calculate P_eq_

As an example of using Equation (8) to calculate *P*_eq_, consider the case where Π_pw_ = 5.

$$P_{eq}^{*}=\frac{1}{1+\Pi_{pw}}=\frac{1}{1+5}=\frac{1}{6}$$

Then *P*_eq_* = 1/6 (≈0.167). Using values for Δ*π* and *P*_C_ as 0.6 MPa and 0.3 MPa, respectively, *P*_eq_ can be calculated from Equation (8) as follows:

$$P_{eq}-P_{C}=\frac{1}{6}\;\left(\Delta \pi -P_{C}\right)\;and\;P_{eq}=\frac{1}{6}\;\left(\Delta \pi -P_{C}\right)+P_{C}=\;\frac{0.3\;MPa}{6}+0.3\;MPa=0.35\;MPa$$

Inspection of Figure 2 shows that when Π_pw_ = 5, *P*_eq_ = 0.35 MPa.

## Appendix E. Derivation of Equation (12)

Beginning with the expression $\Pi_{pw}=\left(\frac{\;\phi_{p}\;}{L_{w}}\right)$, then $L_{w}=\frac{\phi_{p}}{\Pi_{pw}}$. Substituting into Equation (11), the expression for Equation (12) is obtained.

$$v_{s}=\frac{\phi_{p}\;L_{w}}{\phi_{p}+L_{w}}\;\left(∆\pi -P_{C}\right)=\frac{\;\phi_{p}\;\frac{\phi_{p}}{\;\Pi_{pw}}\;}{\phi_{p}\;+\;\frac{\phi_{p}}{\Pi_{pw}}}\;\left(∆\pi -P_{C}\right)=\frac{\;\phi_{p}\;\frac{\phi_{p}}{\;\Pi_{pw}}\;}{\phi_{p}\;\left(1+\frac{1}{\Pi_{pw}}\right)\;}\;\left(∆\pi -P_{C}\right)$$

$$=\frac{\;\frac{\phi_{p}}{\;\Pi_{pw}}\;}{\left(1\;+\;\frac{1}{\Pi_{pw}}\right)\;}\;\left(∆\pi -P_{C}\right)=\frac{\;\frac{\phi_{p}}{\;\Pi_{pw}}\;}{\left(\frac{\Pi_{pw}+\;1}{\Pi_{pw}}\right)\;}\;\left(∆\pi -P_{C}\right)=\frac{\;1\;}{\;\left(\Pi_{pw}+\;1\right)}\;\phi_{p}\;\left(∆\pi -P_{C}\right)$$
